# Inhibition of TERC inhibits neural apoptosis and inflammation in spinal cord injury through Akt activation and p-38 inhibition via the miR-34a-5p/XBP-1 axis

**DOI:** 10.1515/med-2022-0619

**Published:** 2023-01-24

**Authors:** Weiguo Ding, Weixing Xu, Di Lu, Hongfeng Sheng, Xinwei Xu, Bin Xu, Aote Zheng

**Affiliations:** Department of Orthopedics, Tongde Hospital of Zhejiang Province, Hangzhou, Zhejiang Province, 310012, China

**Keywords:** TERC, spinal cord injury, tissues regeneration, inflammation, miR-34a-5p

## Abstract

This study investigated the function of telomerase RNA component (TERC) in spinal cord injury (SCI). SCI models were established in rats via laminectomy and PC-12 cells were treated with lipopolysaccharide (LPS). TERC and miR-34a-5p expressions in cells and rat spinal cords were detected by quantitative reverse transcription polymerase chain reaction, followed by overexpression/knockdown of TERC/miR-34a-5p. Spinal cord histopathological changes were examined via hematoxylin–eosin staining. miR-34a-5p′ relation with TERC and XBP-1 was predicted by TargetScan and checked by dual-luciferase reporter/RNA immunoprecipitation assays. Cell biological behaviors were assessed by Cell counting kit-8, wound healing, Transwell, and flow cytometry assays. XBP-1 and inflammation/apoptosis-related protein expressions were analyzed by western blot. TERC was upregulated and miR-34a-5p was low-expressed in SCI tissues and LPS-induced PC-12 cells. TERC-knockdown alleviated histopathological abnormalities yet upregulated miR-34a-5p in SCI tissues. In LPS-induced PC-12 cells, TERC knockdown promoted cell viability, migration, invasion, and inhibited apoptosis, while TERC overexpression ran oppositely. TERC knockdown downregulated the XBP-1, IL-6, TNF-α, Bax, p-p38/t-p38, and cleaved caspase-9/-3, but upregulated Bcl-2 and p-Akt/t-Akt. TERC targeted miR-34a-5p, which further targeted XBP-1. miR-34a-5p downregulation exerted effects opposite to and offset TERC knockdown-induced effects. TERC knockdown facilitated the regeneration of neuron tissues yet inhibited inflammation in SCI through Akt activation and p-38 inhibition via the miR-34a-5p/XBP-1 axis.

## 1 Introduction

Spinal cord injury (SCI), occurring after high-energy blunt traumas or with low-energy mechanisms, refers to neuronal dysfunction in the spinal cord [1]. At the primary stage of SCI, synaptic connections are lost, signal propagation is disrupted by demyelination and axon damage, and neuron death and progressive tissue degeneration are mechanically induced [2]. These primary events can trigger a secondary cascade in which vascular, inflammatory, and biochemical events develop to further disrupt the neuronal function and aggravate tissue degeneration [2].

Currently, SCI is considered incurable, owing to the great difficulty in nervous tissue regeneration [3]. In normal tissue healing, tissue remodeling is initiated and lasts for a long period of time that includes the inflammatory and proliferative phases of repair after injury [4]. Macrophages, activated during the primary and secondary stages of SCI [2], contribute to the tissue regeneration process through transforming into reparative phenotypes [4]. During macrophage-mediated tissue regeneration, M1-type macrophages produce pro-inflammatory cytokines and promote phagocytosis to accelerate innate immunity-regulated removal of foreign microbes and wound debris, whereas M2-type macrophages inhibit the release of pro-inflammatory cytokines and reactive oxygen species, and secrete immunosuppressive cytokines and chemokines to exert tissue-repairing effects [4]. In contrast, in SCI, the transformation of macrophages into reparative phenotypes is inaptly induced or absent, resulting in maladaptive response in tissue regeneration [4]. Therefore, an approach that positively exploits the mechanism underlying macrophage-mediated normal reparative process may greatly facilitate the recovery from SCI.

Long noncoding RNAs (lncRNAs), constituted by >200 nucleotides, are regulatory transcripts, many of which have been discovered to demonstrate dynamic expression patterns in various pathological processes including SCI [5,6]. Previous studies have recorded that lncRNAs can regulate apoptosis of neuronal cells in acute SCI [7], and viability, migration, and invasion of H_2_O_2_-injuried neuronal cells [8]. These regulatory effects of lncRNAs on diversified diseases can be achieved through the construction of a competing endogenous RNA (ceRNA) network in which lncRNAs competitively bind miRNAs, thereby positively regulating the expression of the target gene of the miRNAs [9]. Human telomerase RNA component (TERC), an essential component of telomerase, is a 451-nucleotide-long lncRNA that can serve as a template for telomere replication [10]. TERC is expressed in telomerase-expressing cells, and also in most terminally differentiated cells, which are telomerase-inactive [11,12,13]. TERC has been found to be able to stimulate the NF-κB pathway and to induce the production of inflammation cytokines [13], critically involving in organ regeneration that is associated with cell proliferation [14]. However, the functional significance and molecular mechanism of TERC in SCI have been rarely investigated yet.

miR-34a has been identified as an inflammation alleviator for SCI [15]. Deng’s study has demonstrated that inhibition of miR-34a-5p, which is downregulated in lipopolysaccharide (LPS)-induced neuronal cells, restrained inflammation suppression caused by inhibition of LINC00665, an upstream lncRNA of miR-34a-5p [16].

Here, this study was set to unveil the function of TERC on the biological behavior of neuronal cells and inflammation responses in SCI, and explore the role of miR-34a-5p in TERC-mediated SCI progression, for providing a novel strategy to accelerate the recovery from SCI.

## 2 Materials and methods

### 2.1 Animal experiment

Twenty-four male Wistar rats (10–12 weeks old, and weighing around 230–250 g) were purchased from the Model Animals Institute of Nanjing University (Nanjing, China), and were maintained at 21–23°C, in 55 ± 5% humidity, under a 12 h:12 h circadian cycle, with free access to food and water. All the rats were randomly assigned to four groups (*n* = 6 per group): Sham group, SCI group, ShNC + SCI group, and ShTERC + SCI group. For SCI induction [15], following anesthetization using 35 mg/kg pentobarbital sodium (P-010, Sigma-Aldrich, USA), the rats in the SCI, ShNC + SCI, and ShTERC + SCI groups were incised at the back posterior to the lower thoracic region. Back muscles were separated to expose the dorsal surface of the spinal cord at T10, after which the lower thoracic cord was transected by using sterile scissors. Lentiviral vectors carrying ShTERC or ShNC (lentivirus-ShTERC/ShNC) were constructed by Genepharma (Shanghai, China). For TERC knockdown, 8 µL of normal saline containing lentivirus-ShTERC/ShNC (10^7^ TU/mL) was injected to each rat in the ShTERC + SCI and ShNC + SCI groups at two sites, 2 mm away from the margin of the head or back ends of the incision and 1 mm away from the central vein of the back. The rats in the Sham group were given anesthetization using 35 mg/kg pentobarbital sodium, without surgery and lentivirus injection. Twelve hours after SCI induction, all the rats were sacrificed by decollation under anesthetization and their spinal cords were harvested.

### 2.2 Hematoxylin–eosin staining

Rat spinal cords were fixed in 4% paraformaldehyde (16005, Sigma-Aldrich, St. Louis, MO, USA) for 24 h, followed by transparentization using xylene (95682, Sigma-Aldrich, USA), dehydration with gradient ethanol, and embedment in paraffin (1496904, Sigma-Aldrich, USA). The paraffin-embedded spinal cords were sliced into 5 μm thick sections by using a slicer (pfm3005E, Dakewe Biotech Co., Ltd, Shenzhen, China), dewaxed by xylene, and rehydrated by gradient ethanol. Then, the sections were stained with hematoxylin (H3136, Sigma-Aldrich, USA) for 7 min, and later differentiated by 1% hydrochloric alcohol (56694, Sigma-Aldrich, USA) for the removal of excessive pigments. After developing blue in weakly alkaline water, the sections were stained with eosin (E4009, Sigma-Aldrich, USA) for 2 min. Following rinse with distilled water, dehydration using gradient ethanol, and transparentization utilizing xylene, the stained sections were observed by an optical microscope (IX71; Olympus, Tokyo, Japan) under ×100 magnification.

### 2.3 Cell culture and treatment

Rat adrenal pheochromocytoma PC-12 cells were purchased from Procell (CL-0412, Wuhan, China), and were cultured in RPMI-1640 media (A4192301, Thermo Fisher, USA) supplemented with 15% horse serum (164215, Procell, China), 5% fetal bovine serum (FBS, F2442, Sigma-Aldrich, USA), and 1% streptomycin–penicillin (V900929, Sigma-Aldrich, USA) at 37°C with 5% CO_2_. LPS ( L7770, Sigma-Aldrich, USA) was used to establish SCI on PC-12 cells [17]. PC-12 cells were treated by LPS at different concentrations (2, 4, 8, and 16 μg/mL) for 12 h.

### 2.4 Cell transfection

miR-34a-5p mimic/inhibitor, and mimic/inhibitor control were purchased from RIBOBIO (miR10000815-1-5/miR20000815-1-5 and miR1N0000001-1-5/miR2N0000001-1-5, Guangzhou, China). ShTERC was constructed with MISSION pLKO.1-puro eGFP shRNA Control plasmids (SHC005, Sigma-Aldrich, USA) and sequence (Forward Oligo: 5′-CCGGTAGCTGTGGGTTCTGTTCTTTCTCGAGAAAGAACAGAACCCACAGCTATTTTTG-3′; Reverse Oligo: 5′-AATTCAAAAATAGCTGTGGGTTCTGTTCTTTCTCGAGAAAGAACAGAACCCACAGCTA-3′) and added in animal experiment, and the empty plasmid was used as shNC. Lipofectamine 3,000 transfection reagents (L3000015, Thermo Fisher, Waltham, MA, USA) were used to transfect the above plasmids into PC-12 cells alone or in combination. Briefly, PC-12 cells were plated at a density of 1 × 10^4^ cells/well in 96-well plates. When the cells reached 80% confluence, the above plasmids (0.2 µg) and lipofectamine 3,000 transfection reagents (0.15 µL) were diluted in both Opti-MEM media (10 µL) (31985062, Thermo Fisher, USA) and P3000 reagents (0.4 µL), and then incubated together at 37°C for 10 min. After incubation, gene–lipid complexes were obtained and used to incubate the cells at 37°C for 24 or 48 h.

### 2.5 Flow cytometry

The apoptosis of PC-12 cells was measured by Annexin V-FITC/PI apoptosis detection kit (40302ES20, Yeasen, Shanghai, China). After transfection and LPS treatment, PC-12 cells were digested in EDTA-free trypsin (T2600000, Sigma-Aldrich, USA), and washed with phosphate buffered saline (PBS; AM9624, Thermo Fisher, USA) thrice. The cells were then resuspended by 1× Binding Buffer to be 1 × 10^6^ cells/mL and added with Annexin V-FITC solution (5 μL) and propidium iodide (PI) solution (10 μL). After 10 min incubation in the dark, apoptotic cells were examined by a flow cytometer (CytoFLEX, Beckman Coulter, Brea, CA, USA).

### 2.6 Cell counting kit (CCK)-8 assay

After transfection and LPS treatment, PC-12 cells were plated in 96-well plates at a density of 5 × 10^3^ cells/well. CCK-8 reagent (96992, Sigma-Aldrich, USA) was added to the cells at a ratio of 1:10, following which cell incubation was conducted at 37°C for 1 h. Cell viability was calculated based on cell absorbance recorded by a microplate reader (ELx808, BioTek, Winooski, VT, USA) at 450 nm.

### 2.7 Wound healing assay

After transfection and LPS treatment, PC-12 cells were plated at a density of 2 × 10^4^ cells/well in 6-well plates. The cells were cultured in serum-free RPMI-1640 until cell monolayers were formed. Subsequently, a gap was scraped by a sterile pipette tip on each monolayer. After scraped cells were removed, the gaps were photographed at 0 and 24 h, and the monolayers were incubated at 37°C for 24 h. Uncovered areas from eight randomly selected fields were observed by an inverted microscope (IX71; Olympus, Tokyo, Japan) under ×100 magnification. The widths of the areas were analyzed using an image analysis system (Wound Healing ACAS, ibidi, Munich, Germany).

### 2.8 Transwell assay

The invasive ability of transfected PC-12 cells was evaluated by Transwell chambers (3428, Corning, NY, USA). Matrigel (356234, Corning, USA) (dilution 1:3) was laid onto the upper chamber. After transfection and LPS treatment, PC-12 cells were suspended by serum-free RPMI-1640 to be 2 × 10^5^ cells/mL, and 100 µL of this cell solution was poured into the upper chamber. RPMI-1640 (600 µL) containing 10% FBS was added into the lower chamber. After 24 h incubation at 37°C, non-invading cells in the upper chamber were removed, following which remanent cells were washed by PBS twice, fixed in 4% paraformaldehyde (P6148, Sigma-Aldrich, USA) and stained with Giemsa (800 µL) (10092013, Thermo Fisher, USA). Stained cells from eight randomly selected fields were observed by an inverted microscope (IX71; Olympus, Tokyo, Japan) under ×200 magnification.

### 2.9 Quantitative reverse transcription polymerase chain reaction (qRT-PCR)

Rat spinal cord tissues were homogenized by a homogenizer (UH-05, Union-Biotech, Shanghai, China). Total RNA and total miRNA from PC-12 cells and rat spinal cord homogenates were extracted by Trizol reagents (15596026, Thermo Fisher, USA) and PureLink miRNA Isolation Kits (K157001, Thermo Fisher, USA), respectively. cDNAs were synthesized via the reverse transcription of the extracted total RNA and total miRNA by SuperScript IV reverse transcriptases (3531295001, Sigma-Aldrich, USA). cDNA amplification was performed with PowerUp SYBR Green Master Mix (A25742, Thermo Fisher, USA) and analyzed by the Real-Time PCR Detection System (CFX Connect, Bio-Rad, Philadelphia, PA, USA), the primers sequence was showed in Table 1. PCR reaction was initiated at the following conditions: 95°C for 10 min, 40 circles of 95°C for 15 s, and 60°C for 60 s. TERC was normalized to GAPDH and miR-34a-5p was normalized to U6. The relative gene expressions were presented by the 2^−ΔΔCt^ method [18].

**Table 1:** Primers used in quantitative reverse transcription polymerase chain reaction for related genes

Gene	Species	Forward	Reverse
TERC	Rat	5′-GGAACTGGTCCCTGAGTTCG-3′	5′-GGTGCACTTCCCACATCTCA-3′
miR-34a-5p	Rat	5′-TGGCAGTGTCTTAGCTGGTT-3′	5′-TGTCGTGGAGTCGGCAATTG-3′
XBP-1	Rat	5′-AGGAGCCTGTAGGACGGAAT-3′	5′-TCCCGTTGCGTCATAAGCTT-3′
GAPDH	Rat	5′-TTCACCACCATGGAGAAGGC-3′	5′-GGCATGGACTGTGGTCATGA-3′
U6	Rat	5′-CTCGCTTCGGCAGCACA-3′	5′-AACGCTTCACGAATTTGCGT-3′

### 2.10 Western blot

Total protein from transfected PC-12 cells was extracted by RIPA Buffer (89900, Thermo Fisher, USA), and quantitated by BCA kit (A53227, Thermo Fisher, USA). Marker (4 μL) (PR1910, Solarbio, Beijing, China) and the extracted protein (40 μg) were separately loaded and subjected to electrophoresis on 10 or 12% SDS-PAGE gel (P0670 or P0672, Beyotime, Shanghai, China), followed by electroblotting onto PVDF membranes (P2438, Sigma-Aldrich, USA). The membranes were blocked by 5% non-fat milk in Tris Buffered Saline with 1% Tween 20 (TBST, TA-125-TT, Thermo Fisher, USA) for 1 h, and incubated with primary antibodies at 4°C overnight, including those against XBP-1 (#40435, 55 kDa, 1:1,000, Danvers, MA), IL-6 (#12912, 24 kDa, 1:1,000), TNF-α (#11948, 25 kDa, 1:1,000), t-p38 (#9212, 40 kDa, 1:1,000), p-p38 (#4511, 43 kDa, 1:1,000), t-Akt (#4691, 60 kDa, 1:1,000), p-Akt (#4060, 60 kDa, 1:2,000), Bcl-2 (#3498, 26 kDa, 1:1,000), Bax (#14796, 20 kDa, 1:1,000), cleaved caspase-9 (#9509, 37 kDa, 1:1,000), cleaved caspase-3 (#9661, 17 kDa, 1:1,000), and GAPDH (#5174, 37 kDa, 1:1,000) purchased from Cell signaling technology in USA. The membranes were then washed with TBST and incubated with secondary antibody Goat anti-Rabbit IgG (A32731, 1:10,000, Thermo Fisher, USA). Immunoreactive bands were visualized by enhanced chemiluminescence reagent kit (WP20005, Thermo Fisher, USA) on an imaging device (iBright CL750, Thermo Fisher, USA) and analyzed quantitatively by ImageJ software (1.52 s version, National Institutes of Health, Bethesda, MA, USA).

### 2.11 Dual-luciferase reporter assay

The binding sites between TERC and miR-34a-5p and between miR-34a-5p and XBP-1 was performed using TargetScan V7.2 (http://www.targetscan.org/mamm_31/). Sequences of TERC (wild type (wt): 5′-TTCTCCGGAGGCACCCACTGCCA-3′, mutant type (mut): 5′-TTCTCCGGAGGCACCCACGACCA-3′), and XBP-1 (wt: 5′-GCTTTCATC-CAGCCACTGCCC-3′, mut: 5′-GCTTTCATC-CAGCCCGTGCCC-3′) were separately cloned to pmirGLO vectors (E1330, Promega, Madison, WI, USA) for the obtainment of reporter plasmids. PC-12 cells were cultured in 12-well plates at a density of 1 × 10^7^ cells/well. When the cells reached a 70% confluence, the cells were co-transfected with the reporter plasmids (100 ng) and miR-34a-5p mimic/miR-NC (100 ng) by Lipofectamine 3,000 reagents. After 48 h of transfection at 37°C, dual luciferase reporter assay was performed with a dual-luciferase reporter assay system (E1980, Promega, USA). The firefly luciferase activity, which indicated the binding specificity, was normalized to *renilla* luciferase activity, and measured by a luminometer (GloMax®20/20, Promega, USA).

### 2.12 RNA immunoprecipitation (RIP) assay

Binding relationship between miR-34a-5p and TERC or XBP-1 was validated by using Magna RIP Kits (17-704, Sigma-Aldrich, USA). Cells were treated with lysis buffer (P0013B, Beyotime, China) on ice, followed by a 10 min centrifugation at 10,000× *g* at 4°C. After that, supernatant was obtained and was precleared with magnetic beads. Then, the precleared supernatant was re-suspended in RIP Wash Buffer and incubated with protein A/G magnetic beads bound with anti-Argonaute2 (Ago2) antibody (ab32381, 1:50, Abcam, UK) or monoclonal antibody IgG (ab172730, 1:100, Abcam, UK) overnight at 4°C. Afterwards, the precipitate was harvested and digested with Proteinase K and qRT-PCR was used for assessing the enrichment of TERC or XBP-1.

### 2.13 Statistical analysis

Statistical analyses were performed with Graphpad prism (version 8.0, GraphPad Software Inc., San Diego, CA, USA). All data were obtained from independent experiments performed in triplicate and expressed as mean value ± standard deviation (SD). Differences among multiple groups were analyzed by one-way analysis of variance (ANOVA), and those between two groups were analyzed by independent *t*-test, followed by Tukey’s *post-hoc* test. *P* < 0.05 was regarded statistically significant.


**Ethics statement:** All animal experiments were performed in accordance with the guidelines of the China Council on Animal Care and Use. This study was approved by the Committee of Experimental Animals of Zhejiang Academy of Traditional Chinese Medicine (approval number: ZATCM Animal Ethic Review no. [2019]048). Every effort was made to minimize pain and discomfort to the animals. The animal experiments were performed in Zhejiang Academy of Traditional Chinese Medicine.

## 3 Results

### 3.1 TERC was overexpressed but miR-34a-5p was lowly expressed in the spinal cord of SCI rats, and knockdown of TERC promoted the miR-34a-5p expression and alleviated histopathological abnormalities

**Figure 1:**
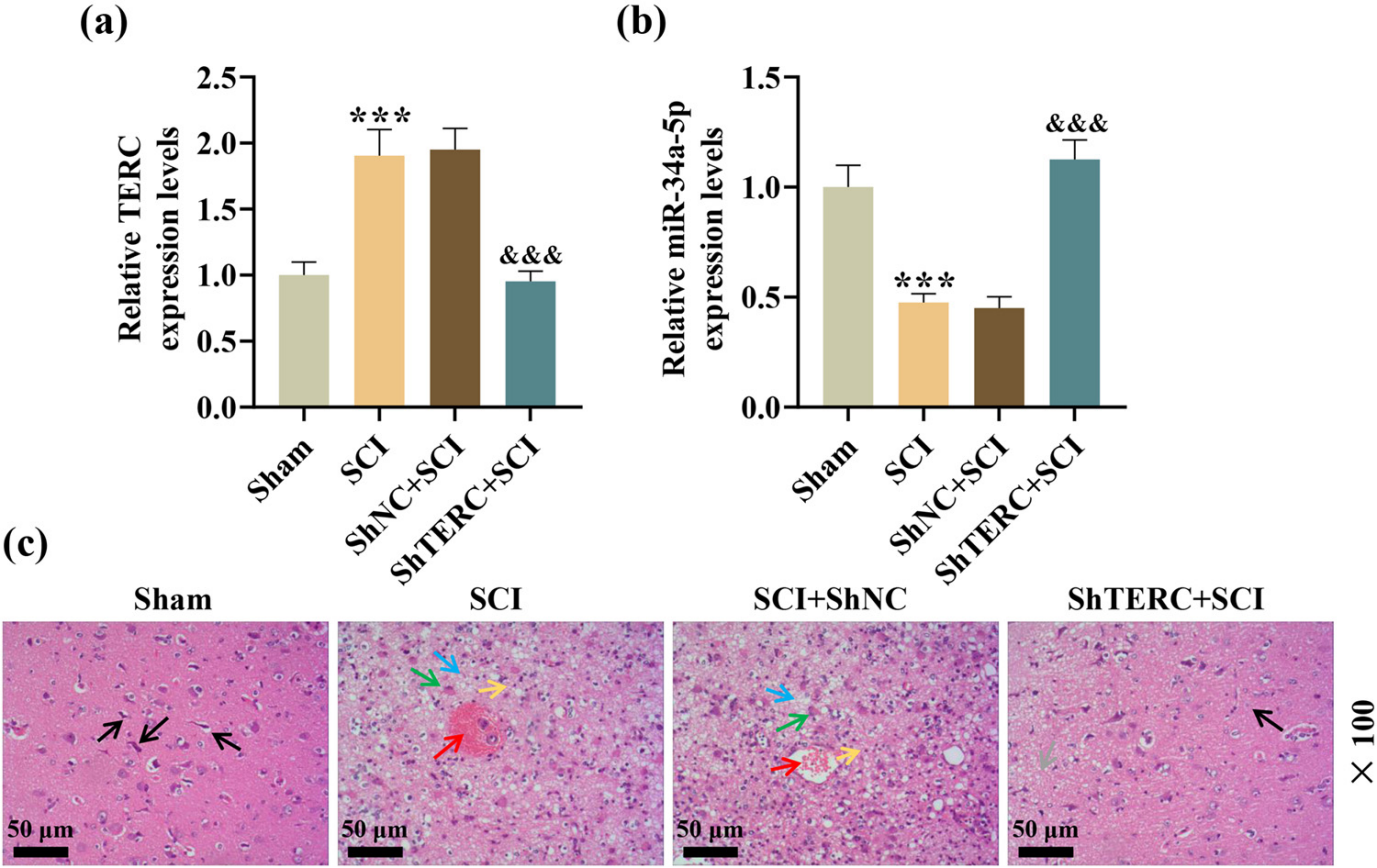
Knockdown of TERC, which was upregulated in SCI, revoked miR-34a-5p downregulation, and alleviated histopathological abnormalities in the spinal cord of SCI rats. (a and b) The expressions of TERC and miR-34a-5p in the spinal cords of SCI rats were analyzed by qRT-PCR. (c) Spinal cord histopathological changes were examined via hematoxylin–eosin staining (magnification: ×100; scale: 50 µm; black arrows: a basically complete and regular organizational structure; blue arrows: a relatively disorganized organizational structure; yellow arrows: pyknosis and degeneration of the nucleus; green arrows: obviously loose and edematous interstitium; red arrows: local congestion and bleeding; gray arrows: slightly loose interstitium). ^***^ *P* or ^&&&^ *P* < 0.001; ^*^ vs Sham; ^&^ vs ShNC + SCI.

TERC has been found to promote cellular inflammation [13]. A significantly upregulated TERC was detected in the spinal cord tissues of SCI rats through qRT-PCR, compared to the TERC level in the spinal cord tissues of the sham rats (Figure 1a). miR-34a-5p has been found to be downregulated in SCI [16]. Consistently, qRT-PCR analysis showed that miR-34a-5p level was downregulated in the spinal cord tissues of SCI rats, compared with that in the spinal cord tissues of the sham rats (Figure 1b). To explore the role of TERC on SCI-associated miR-34a-5p downregulation and histopathological changes, lentivirus-shTERC was employed. Injection of lentivirus-shTERC successfully downregulated TERC, and led to an increased level of miR-34a-5p (Figure 1a and b). Histopathological examination by hematoxylin–eosin staining illustrated that the sham rats had structurally dense and regular spinal cord tissues, which were enriched with neuronal cells showing large cell bodies, delicate nuclear staining and good morphological differentiation and presented dense and uniform interstitium with no inflammatory cell infiltration, whereas structurally relatively disordered spinal cord tissues, which harbored neuronal cells with eosinophilic change, cell body shrinkage, nucleus pyknosis, and degeneration and showed obviously loose and edematous interstitium with local congestion and bleeding and more inflammatory cell infiltration, were observed in the SCI rats; notably, injection of lentivirus-shTERC pronouncedly alleviated the above pathological conditions in the spinal cord tissues of the SCI rats, as following TERC knockdown, the SCI rats presented complete and regular spinal cord tissues with mild loose interstitium, a small number of interstitium-infiltrating inflammatory cells, and abundant intercellular neuronal cells that were mostly well differentiated and hardly showed pyknotic changes (Figure 1c).

### 3.2 TERC level was upregulated while miR-34a-5p level was downregulated in LPS-induced PC-12 cells

**Figure 2:**
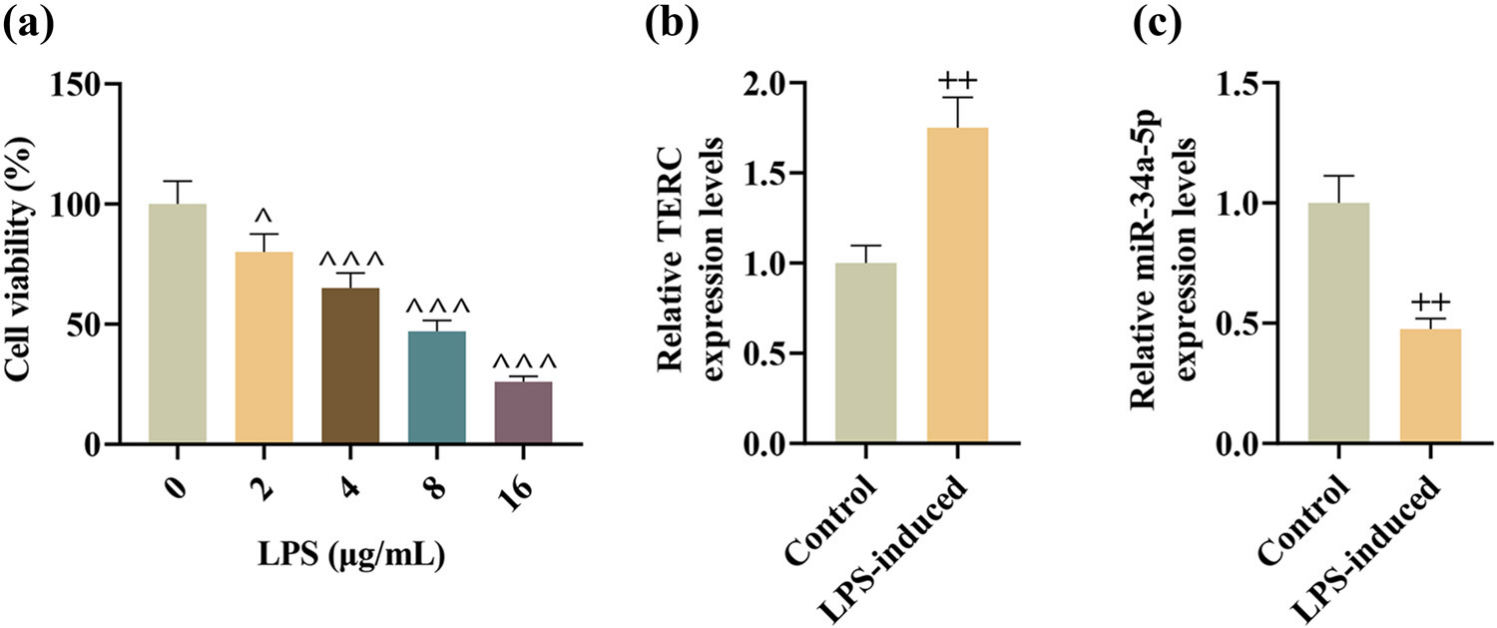
TERC level was upregulated while miR-34a-5p level was downregulated in LPS-induced PC-12 cells. (a) The viability of PC-12 cells after treatment with different concentrations of LPS was measured by CCK-8 assay. (b and c) The expressions of TERC and miR-34a-5p in LPS-induced PC-12 cells were analyzed by qRT-PCR. ^^^ *P* < 0.05, ^++^ *P* < 0.01, ^^^^^ *P* < 0.001; ^^^ vs 0; ^+^ vs Control.

Next, SCI models *in vitro* were established with LPS-induced PC12 cells. As shown in CCk-8 assay, treatment with LPS concentration-dependently decreased the viability of PC-12 cells (Figure 2a). Given that the viability of PC-12 cells was decreased by half after the PC-12 cells were treated with LPS of 4 μg/mL, 4 μg/mL was selected as the optimal concentration for the establishment of SCI models *in vitro*. QRT-PCR analysis showed that TERC level was upregulated, but miR-34a-5p level was downregulated in LPS-induced PC-12 cells, compared to those in the control cells (Figure 2b and c).

### 3.3 TERC regulated the viability, migration, invasion, and apoptosis of LPS-induced PC-12 cells

**Figure 3:**
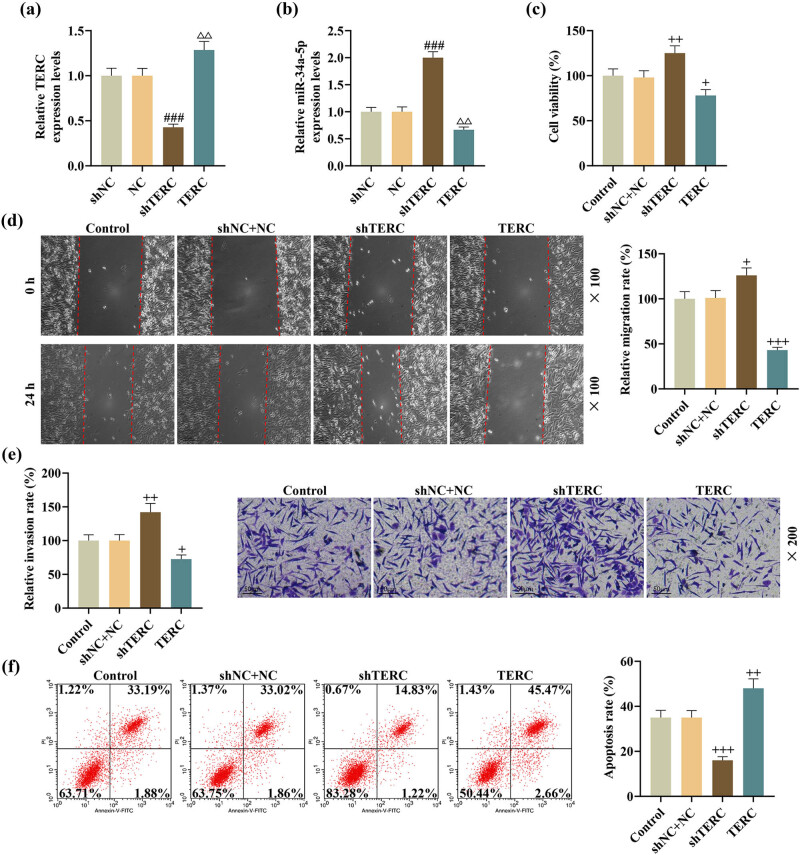
TERC regulated the viability, migration, invasion, and apoptosis of LPS-induced PC-12 cells. (a and b) The expressions of TERC and miR-34a-5p in shTERC/TERC overexpression plasmid-transfected PC-12 cells after LPS treatment were analyzed by qRT-PCR. (c) The viability of shTERC/TERC overexpression plasmid-transfected PC-12 cells after LPS treatment was measured by CCK-8 assay. (d) The migratory ability of shTERC/TERC overexpression plasmid-transfected PC-12 cells after LPS treatment was evaluated by wound healing assay (magnification: ×100; scale: 100 µm). (e) The invasive ability of shTERC/TERC overexpression plasmid-transfected PC-12 cells after LPS treatment was assessed by Transwell assay (magnification: ×200; scale: 50 µm). (f) The apoptosis of shTERC/TERC overexpression plasmid-transfected PC-12 cells after LPS treatment was detected by flow cytometry. ^+^ *P* < 0.05, ^ΔΔ^ *P* or ^++^ *P* < 0.01, ^###^ *P* or ^+++^ *P* < 0.001; ^#^ vs shNC; ^Δ^ vs NC; ^+^ vs shNC + NC.

ShTERC and TERC overexpression plasmids were employed to investigate the effect of TERC on the biological behaviors in SCI *in vitro*. Transfection of ShTERC or TERC overexpression plasmids successfully led to knockdown or overexpression of TERC, and ShTERC or TERC overexpression promoted or inhibited the expression of miR-34a-5p (Figure 3a and b). As displayed in cellular behavior assessment based on CCK-8, wound healing, and Transwell assays, TERC knockdown increased the viability of LPS-induced PC-12 cells, as well as the migration and invasion by LPS-induced PC-12 cells, whereas TERC overexpression exerts effects opposite to those of TERC knockdown (Figure 3c–e). Following the detection of flow cytometry, the apoptosis of LPS-induced PC-12 cells was found to be inhibited by TERC knockdown, yet enhanced with TERC overexpression (Figure 3f).

### 3.4 Downregulation of miR-34a-5p, a target of TERC, offset TERC knockdown-induced effects on the viability, migration, and invasion of LPS-induced PC-12 cells

**Figure 4:**
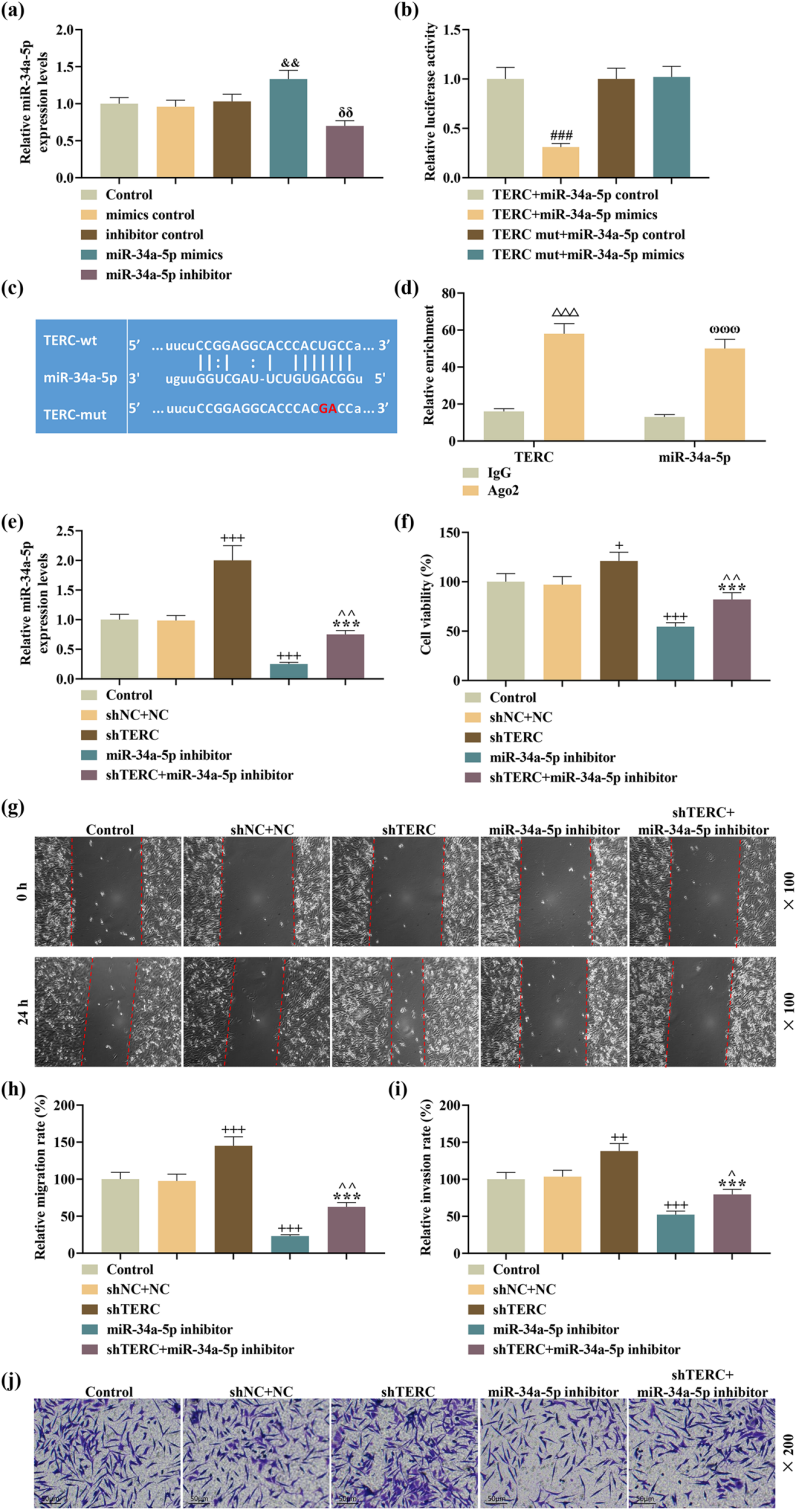
Downregulation of miR-34a-5p, a target of TERC, offset TERC knockdown-induced effects on viability, migration, and invasion of LPS-induced PC-12 cells. (a) The expression of miR-34a-5p in miR-34a-5p mimic/inhibitor-transfected PC-12 cells after LPS treatment was analyzed by qRT-PCR. (b–d) The interaction between TERC and miR-34a-5p was checked by (b) dual-luciferase reporter assay, predicted by (c) TargetScan V7.2, and reaffirmed by (d) RIP assay. (e) The expression of miR-34a-5p in miR-34a-5p inhibitor and/or shTERC-transfected PC-12 cells after LPS treatment was determined by qRT-PCR. (f) The viability of miR-34a-5p inhibitor and/or shTERC-transfected PC-12 cells after LPS treatment was measured by CCK-8 assay. (g and h) The migratory ability of miR-34a-5p inhibitor and/or shTERC-transfected PC-12 cells after LPS treatment was evaluated by wound healing assay (magnification: ×100; scale: 100 µm). (i and j). The invasive ability of miR-34a-5p inhibitor/shTERC-transfected PC-12 cells after LPS treatment was assessed by Transwell assay (magnification: ×200; scale: 50 µm). ^+^ *P* or ^^^ *P* < 0.05; ^&&^ *P* or ^δδ^ *P* or ^++^ *P* or ^^^^ *P* < 0.01, ^###^ *P* or ^+++^ *P* or ^***^ *P* < 0.001, ^△△△^ *P* < 0.001; ^ωωω^ *P* < 0.001; ^&^ vs mimics control; ^δ^ vs inhibitor control; ^#^ vs TERC + miR-34a-5p control; ^+^vs shNC + NC; ^*^ vs shTERC; ^^^ vs miR-34a-5p inhibitor; △ vs IgG in TERC; ^ω^ vs IgG in miR-34a-5p.

The role of miR-34a-5p in TERC-regulated progression of SCI *in vitro* was investigated. A successful upregulation or downregulation of miR-34a-5p level was caused by transfection of miR-34a-5p mimic or inhibitor (Figure 4a). Dual-luciferase reporter assay revealed that the luciferase activity of LPS-induced PC-12 cells containing sequences of TERC-wt was decreased by upregulation of miR-34a-5p (Figure 4b). Analysis based on bioinformatics tools showed that there were sites that can bind miR-34a-5p to TERC (Figure 4c). Moreover, RIP assay was conducted to verify the targeting relationship between TERC and miR-34a-5p. It was found that the addition of Ago2 antibody caused a co-enrichment of miR-34a-5p and TERC in PC-12 cells (Figure 4d). TERC knockdown was demonstrated to upregulate miR-34a-5p level and reverse miR-34a-5p inhibitor-induced downregulation of miR-34a-5p. In turn, miR-34a-5p inhibitor offset TERC knockdown-induced upregulation of miR-34a-5p in LPS-induced PC-12 cells (Figure 4e). Through CCK-8, wound healing, and Transwell assays, we observed that miR-34a-5p downregulation decreased the viability, migration and invasion of LPS-induced PC-12 cells. Moreover, miR-34a-5p downregulation offset TERC knockdown-induced promotion on the cell viability, migration and invasion (Figure 4f–j). In addition, those effects induced by miR-34a-5p inhibitor on these biological behaviors were reversed following the knockdown of TERC (Figure 4f–j).

### 3.5 Knockdown of TERC inhibited apoptosis, inflammation, and p38 activation, yet activated Akt in LPS-induced PC-12 cells via the miR-34a-5p/XBP-1 axis

**Figure 5:**
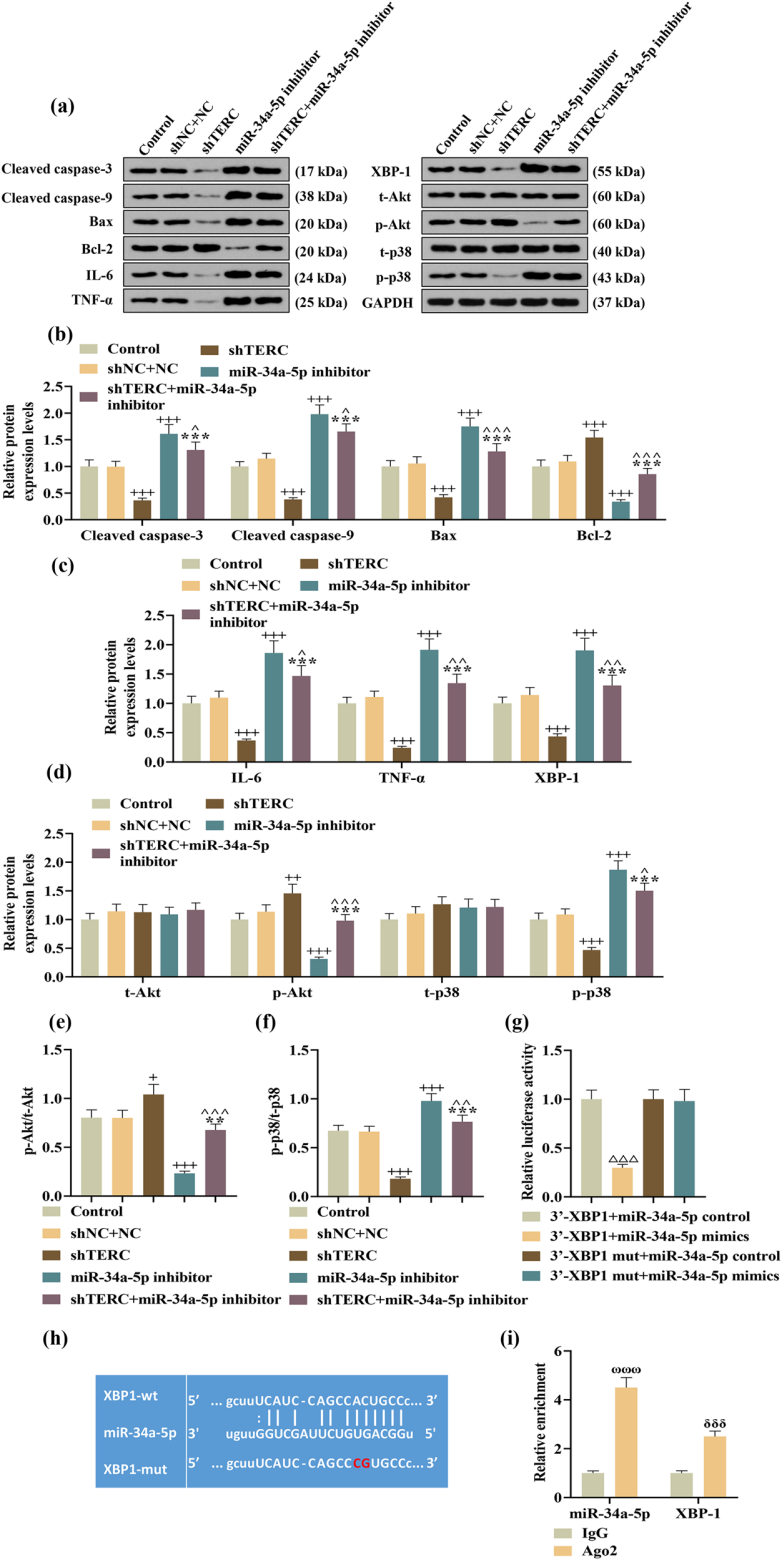
Knockdown of TERC inhibited apoptosis, inflammation, and p38 activation yet activated Akt in LPS-induced PC-12 cells via the miR-34a-5p/XBP-1 axis. (a–f) The expressions of cleaved caspase-3, cleaved caspase-9, Bcl-2, Bax, IL-6, TNF-α, XBP-1, t-Akt, p-Akt, t-p38, p-p38, p-Akt/t-Akt, and p-p38/t-p38 in miR-34a-5p inhibitor and/or shTERC-transfected PC-12 cells after LPS treatment were analyzed by Western blot, with GAPDH serving as a control gene. (g–i). The interaction between miR-34a-5p and XBP-1 was checked by (g) dual-luciferase reporter assay, predicted by (h) TargetScan V7.2, and reaffirmed by (i) RIP assay. ^+^ *P* or ^^^ *P* < 0.05; ^^^^ *P* or ^++^ *P* < 0.01, ^+++^ *P* or ^***^ *P* or ^^^^^ *P* or ^ΔΔΔ^ *P*or < 0.001, ^δδδ^ *P* < 0.001; ^ωωω^ *P* < 0.001; ^+^vs shNC + NC; ^*^vs shTERC^; ^^vs miR-34a-5p inhibitor; ^Δ^vs 3′-XBP1 + miR-34a-5p control; ^ω^vs IgG in miR-34a-5p; ^δ^vs IgG in XBP-1.

Finally, western blot analysis revealed that in LPS-induced PC-12 cells, the expressions of proapoptotic cleaved caspase-3, cleaved caspase-9, and Bax, together with the expressions of Akt pathway-related factor p-p38 and p-p38/p38, accompanied with the expressions of inflammation-related IL-6, TNF-α, and XBP-1, were all decreased by TERC knockdown, but increased by miR-34a-5p downregulation (Figure 5a–f). Meanwhile, the expressions of antiapoptotic Bcl-2 and the expressions of Akt pathway-related factor p-Akt and p-Akt/Akt were all increased by TERC knockdown, but decreased by miR-34a-5p downregulation (Figure 5a–f). Moreover, there existed a mutually antagonistic mechanism between the effects exerted by TERC knockdown and those by miR-34a-5p downregulation on the expressions of abovementioned proteins (Figure 5a–f). Furthermore, dual-luciferase reporter assay revealed that upregulation of miR-34a-5p induced decreases in the luciferase activity of LPS-induced PC-12 cells containing sequences of XBP-1-wt (Figure 5g), which suggested that XBP-1 was directly targeted by miR-34a-5p. The existence of this targeting relation was further reinforced by a bioinformatics tool-based prediction, which displayed miR-34a-5p had binding sites complementary to the sites on XBP-1 (Figure 5h), and RIP assay, which showed that miR-34a-5p and XBP-1 were co-enriched after the addition of Ago2 antibody (Figure 5i).

## 4 Discussion

SCI is a devastating condition that leads to a series of problems, including neuropathic pain, sensory, and motor dysfunction and even paralysis [19]. The pathophysiological process of SCI is associated with initial mechanical trauma-caused primary injury and secondary damage, which involves inflammation, severe neuron cell death and tissue degeneration [20]. Current treatment strategies for SCI mainly focus on tissue regeneration, which includes axon regeneration and formation of new “neural relays” at the injury sites [21]. However, these strategies take time to exert a reparative effect and lack of prompt treatment for the secondary injury prevents SCI survivors from a satisfactory recovery from SCI.

Studies based on microarray and high-throughput sequence technologies have identified a copious number of lncRNAs that are differentially expressed after SCI [6]. These lncRNAs are revealed to act as diagnostic and therapeutic biomarkers for SCI [7]. Moreover, the functional significance of these lncRNAs has been demonstrated by their regulatory roles on neuron apoptosis, neuronal differentiation, migration of spinal cord progenitor cells, microglial inflammatory response and neuron tissue regeneration [7,22,23,24]. TERC is a lncRNA that can promote cellular inflammatory response though activating the NF-κB pathway and inducing the production of inflammation cytokines [13]. A transcriptomic analysis conducted by Ding et al. showed that TERC was among the downregulated lncRNAs within the transected T10 spinal cord segments of SCI rat [25]. Probably due to the different sampling time between this study and our study, our study discovered that TERC level was upregulated in SCI models *in vitro*. Later, this upregulated expression pattern of TERC in SCI was found consistent with the expression pattern of BDNF-AS that had a pro-SCI role [7]. Then, the pro-SCI role of TERC was confirmed, as evidenced by TERC knockdown-induced alleviation on SCI-related pathological changes. Alleviated pathological changes in the spinal cords are indicative of improved tissue regeneration in SCI [23]. Promotion of cell proliferation, migration, and invasion at SCI-related substantial postinjury cystic cavities are critically important for the support of tissue regeneration [26,27]. Our study revealed that in LPS-induced SCI models *in vitro*, TERC expression levels were negatively correlated with cell viability, migration, and invasion, but positively related to cell apoptosis, which reaffirmed the role of TERC to promote neuron tissue regeneration at the cellular level. Furthermore, release of proinflammatory cytokines and activation of proapoptotic signaling in the secondary injury cascade of SCI can severely impede tissue regeneration [26]. Li’s study has mirrored that the enhancement in LPS-induced apoptosis of PC12 cells is accompanied by the cleavage of caspase-3 and 9 as well as the release of proinflammatory cytokines, IL-6 and TNF-α [17]. Bcl-2 is an anti-apoptotic protein, the level of which is downregulated during apoptosis [28], and the Bcl-2 family members Bax and Bak are activated to induce the release of pro-apoptotic proteins, which can further activate caspase-9 [29]. Activated caspase-9 then directly cleaves and activates caspase-3 [29], and is in turn cleaved by caspase 3 at Asp330 [30]. Meanwhile, Bcl-2 has been found to exert a neuroprotective role in traumatic brain injury [31]. Moreover, IL-6, synthesized at the initial stage of inflammation, can induce an extensive range of acute phase proteins such as C-reactive protein, serum amyloid A, fibrinogen, and hepcidin, and inhibit the production of fibronectin, albumin, and transferrin [32]. TNF-α, one of the most important pro-inflammatory cytokines, participates in a series of pro-inflammatory changes including vasodilatation, edema formation, leukocyte adhesion to epithelium, blood coagulation, and oxidative stress [33]. High secretion of both IL-6 and TNF-α are evidence of M1-type macrophages during inflammation [4]. XBP-1, a transcription factor responsible for generating a highly developed endoplasmic reticulum, can facilitate the production of inflammatory mediators, expanding the secretory pathway [34]. Similar to the results in the study by Li et al. [17], our study showed that TERC knockdown inhibited the production of IL-6, TNF-α, and XBP-1, and decreased the levels of Bax, cleaved caspase-3 and 9, but increased Bcl-2 expression, indicating that TERC knockdown exerted anti-inflammatory and anti-apoptosis effects during SCI.

Through microarray analysis, changes in multiple molecular pathways have been identified after SCI [35]. Among these pathways, P13K/AKT is a pro-survival pathway, activation of which exerts a neuroprotective effect on SCI-related inflammation and apoptosis in spinal cord neural tissues [36,37]. Besides, p38 MAPK is a participant of the MAPK cascade pathway, which is involved in various cellular functions, and p38 activation, characterized by increased p-p38/t-p38, contributes to inducible nitric oxide synthase (iNOS)-induced spinal neuron apoptosis and death [38]. Conforming with the activation pattern of these pathways in the above studies, we detected Akt activation and p38 inhibition induced by TERC knockdown in SCI-related neuron cells.

Furthermore, toll-like receptor 4 (TLR4) is an important player in innate immune and inflammatory responses [39]. After inflammation in the central nervous system, TLR4 is expressed in microglia and astrocyte [40]. TLR4 can activate NF-κB, which further induces transcription of many proinflammatory genes, exacerbating neuronal death and dysfunction [37]. Earlier research has revealed that miR-34a can inhibit the HMGB-1/TLR4 signaling pathway to alleviate inflammation and apoptosis of neuron cell after SCI [15]. Chen’s study has also found that PI3K/AKT signaling activation and suppression of TLR4-mediated inflammatory responses are simultaneously implicated in the improvement of functional recovery after SCI [41]. These studies suggest that miR-34a may also regulate AKT signaling to alleviate SCI. Based on the above suggestion and our finding that TERC knockdown induced Akt activation to alleviate SCI, we hypothesized that miR-34a participated in TERC knockdown-mediated alleviation on SCI. In line with our hypothesis, we subsequently found that miR-34a-5p, whose level was downregulated in SCI spinal cord tissues as reported previously [16], was upregulated after the knockdown of TERC and was identified as a target gene of TERC. Previously, miR-34a-5p has been reported to inhibit proliferation, migration, or invasion in HPV-infected human epidermal keratinocytes [42] and arterial smooth muscle cells under hypoxia [43]. Contrary to these, our experiment *in vitro* showed that miR-34a-5p inhibition inhibited these cell phenotypes of LPS-induced PC-12 cells, and our result is consistent with the role of miR-34a-5p in SH-SY5Y cells and human 293 T cells [44]. Moreover, in our study, miR-34a-5p inhibition was shown to offset the aforementioned effects induced by TERC knockdown in SCI *in vitro*.

Finally, in our study, bioinformatics prediction and dual-luciferase reporter assay analysis revealed that XBP-1 was targeted as a downstream mRNA by miR-34a-5p. Previous research has recorded that XBP-1 downregulating is involved in the inhibitory effect of Valproate or Icariin on endoplasmic reticulum stress-induced neuron apoptosis and it is accompanied by AKT activation [45,46], which suggests that XBP-1 downregulation contributes to neuron apoptosis reduction and negatively relates to Akt activation in this process. Besides, p38 inactivation inhibited iNOS-induced neuron apoptosis [38]. Although currently there are few studies except ours investigating the role of p38 together with that of XBP-1 in SCI, based on this finding and our result that XBP-1 was downregulated in the mitigation of the neuron damage in SCI, we surmise that XBP-1 downregulation is positively related to p38 inhibition in SCI alleviation. The above suggestion and surmise are in line with our finding which demonstrated that XBP-1 was downregulated, p38 was inhibited, and AKT was activated by knockdown of the TERC/miR-34a-5p axis in SCI *in vitro*. Also, this XBP-1 downregulation co-occurred with the inhibition of abovementioned pro-apoptotic proteins. On account of these, we proposed the role of a TERC/miR-34a-5p/XBP-1 ceRNA regulatory network in regulating SCI inflammation. Accordingly, more studies should be set on the role of XBP-1 in TERC-mediated SCI.

In addition, there is a limitation of the current study, as we just examined the effects of TERC knockdown on these events at the cellular level. Therefore, in our subsequent study, the effect of TERC knockdown on these events will be investigated in SCI rats via some neurobehavior tests.

## 5 Conclusion

In conclusion, the present study demonstrated that TERC is highly expressed in SCI, and inhibition of the TERC/miR-34a-5p/XBP-1 ceRNA regulatory network inhibits neural apoptosis and inflammation in SCI.
